# Draft Genome Sequences of 3 Strains of Apilactobacillus kunkeei Isolated from the Bee Gut Microbial Community

**DOI:** 10.1128/MRA.00088-21

**Published:** 2021-04-01

**Authors:** Julien Crovadore, Romain Chablais, François Raffini, Bastien Cochard, Martine Hänzi, François Gérard, Karl Kristian Jensen, François Lefort

**Affiliations:** a Plants and Pathogens Group, Research Institute Land Nature and Environment, HEPIA, HES-SO University of Applied Sciences and Arts Western Switzerland, Jussy, Geneva, Switzerland; b Boswellia SARL, Châteauneuf-de-Galaure, France; c New Nordic HealthBrands AB, Malmö, Sweden; Portland State University

## Abstract

Apilactobacillus kunkeei is a fructophilic lactic acid bacterium found in fructose-rich environments such as flowers, fruits, fermented food, honey, and honeydew, as well as in the guts of fructose-feeding insects. We report here the draft genome sequences of three Apilactobacillus kunkeei strains isolated from the gut microbial community of three honeybees.

## ANNOUNCEMENT

The honeybee digestive track microbiota is dominated by *Lactobacillus* and *Bifidobacterium* species (1, 2), including Apilactobacillus kunkeei (3), a fructophilic and facultative anaerobic Gram-positive rod-shaped bacterium. First identified in damaged grapes (4), it was also found to be an important bacterial component of the bacterial flora in honey, honeydew honey, royal jelly, and pollen from diverse sources (2, 5, 6). It plays a protective role against bee pathogens (7). Present in the gut microbiota of the nine known *Apini* species (8), it might potentially control pathogens in bees and humans (6, 7, 9). These three *A. kunkeei* strains were isolated by the dissection of 3 Apis mellifera individuals, picked in the vicinity of the laboratory. The guts, from foregut to hindgut, were removed from the bees and opened with a sterile scalpel. The gut contents were plated onto MRS plus fructose (2% [wt/vol]) agar medium (10). Subculturing was carried out until isolates were obtained. Pure cultures were grown at 30°C for 48 h for DNA extraction according to a modified cetyltrimethylammonium bromide (CTAB) protocol (11). These strains were identified as *A. kunkeei* by sequencing the 16S amplicons produced with primers 27F and 1492R (12) and comparing them to GenBank sequences (13). Sequencing libraries were prepared with the TruSeq Nano DNA PCR-free library preparation kit (Illumina). Whole-genome sequencing was carried out within one Illumina MiniSeq run with a 2 × 151-bp paired-end read length, using a MiniSeq high-output kit, which provided genome coverage in the range of 219.8× to 361.9×. The Illumina conversion software bcl2fastq2 version 2.20 was automatically run through the MiniSeq local run manager set with default parameters in order to trim the adapters and to demultiplex the samples based on their respective indices. The sequencing quality of the MiniSeq run was high, with 92% of the reads above the quality Phred score of 30. The reads were evaluated for quality, adaptor contamination, and the presence of Ns using FastQC version 0.11.9 (14). The genomes were assembled with the SPAdes version 3.10 genome assembler (15), yielding between 45 and 54 contigs (≥200 bp), and assessed with QUAST (16). The genome lengths varied between 1,485,566 and 1,548,283 bp, within a GC content range of 35.47% to 36.57%. These results are congruent with the literature for fructophilic lactobacilli. These bacteria would have undergone a reductive evolution (17, 18), leading to much smaller genomes (1.42 to 1.55 Mb) than those in other lactobacilli (2.46 Mb on average). The Prokaryotic Genome Automatic Annotation Pipeline (PGAP) (19) and RAST version 2.0 were used to carry out automated gene annotation (20). Using PlasmidFinder (21) and plasmidSPAdes (22), no plasmids were detected. No phage, prophage, or transposon sequences were found in the annotated genomes, in contrast to previous reports (18). Neither annotation found any genes for motility, photosynthesis, or nitrogen metabolism. The numbers of coding sequences were similar to previous reports for fructophilic lactobacilli (17, 18). No toxin or virulence genes or pathogenicity islands were detected through annotation. Putative resistance genes to the fluoroquinolone antibiotic class were found in all genomes, with strain NN5 also containing putative resistance genes to beta-lactamase. These three genome sequences will add to the knowledge of this species within a local bee population.

### Data availability.

This genome-sequencing project has been deposited in the NCBI Sequence Read Archive (SRA) and genome databases. The GenBank, SRA, BioProject, and BioSample accession numbers for the three individual strains are given in Table 1, along with their genome statistics.

**TABLE 1:** Summary of genome sequence statistics for the 3 Apilactobacillus kunkeei strains

Genome characteristic	Data for Apilactobacillus kunkeei strain:		
	UASWS1867-NN5	UASWS1868-NN17	UASWS1870-NN20
Total length (bp)	1,485,566	1,548,283	1,504,698
GC content (%)	36.57	35.47	36.37
No. of CDS^a^	1,338	1,384	1,341
No. of tRNAs	61	65	64
No. of rRNAs	6	4	5
Sequencing yield (Mbp)	326.56	435.83	544.49
Final coverage (×)	219.8	281.5	361.9
SRA accession no. for raw reads	SRX5823656	SRX5823655	SRX5823657
No. of contigs	46	45	54
*N*_50_ (bp)	78,107	135,180	55,820
GenBank accession no. for genome	VBSD00000000	VBSE00000000	VBSF00000000
BioProject accession no.	PRJNA542049	PRJNA542050	PRJNA542054
BioSample accession no.	SAMN11607713	SAMN11607714	SAMN11607716

a CDS, coding sequences.
